# Behavioral Disorders of Spatial Cognition in Patients with Mild Cognitive Impairment Due to Alzheimer’s Disease (The BDSC-MCI Project): Ecological Validity of the Corsi Learning Suvra-Span Test

**DOI:** 10.3390/jpm14050539

**Published:** 2024-05-17

**Authors:** Davide Maria Cammisuli, Gloria Marchesi, Virginia Bellocchio, Edoardo Nicolò Aiello, Barbara Poletti, Federico Verde, Vincenzo Silani, Nicola Ticozzi, Stefano Zago, Teresa Difonzo, Valeria Isella, Simone Pomati, Valentina Granese, Benedetta Vignati, Lorenzo Augusto Prete, Gianluca Castelnuovo

**Affiliations:** 1 Department of Psychology, Catholic University, 20123 Milan, Italy; gloria.marchesi03@icatt.it (G.M.); gianluca.castelnuovo@auxologico.it (G.C.); 2 Catholic University, 20123 Milan, Italy; virginiabellocchio12@gmail.com (V.B.); valentina.granese@unicatt.it (V.G.); benedetta.vignati01@icatt.it (B.V.); lorenzoaugusto.prete@unicatt.it (L.A.P.); 3 Department of Neurology and Laboratory of Neuroscience, IRCCS Istituto Auxologico Italiano, 20149 Milan, Italy; e.aiello@auxologico.it (E.N.A.); b.poletti@auxologico.it (B.P.); f.verde@auxologico.it (F.V.); vincenzo@silani.com (V.S.); n.ticozzi@auxologico.it (N.T.); 4 Department of Oncology and Hemato-Oncology, Università Degli Studi di Milano, 20133 Milan, Italy; 5 Department of Pathophysiology and Transplantation, Dino Ferrari Center, University of Milan, 20122 Milan, Italy; 6 Fondazione IRCCS Ca’ Granda, Ospedale Maggiore Policlinico, University of Milan, 20122 Milan, Italy; stefano.zago@unimi.it (S.Z.); teresa.difonzo@policlinico.mi.it (T.D.); 7 Department of Neurology, School of Medicine, University of Milano-Bicocca, 20126 Milan, Italy; valeria.isella@unimib.it; 8 Milan Centre for Neurosciences, 20133 Milan, Italy; 9 Neurology Unit, Luigi Sacco University Hospital, 20157 Milan, Italy; simone.pomati@asst-fbf-sacco.it; 10 IRCCS Istituto Auxologico Italiano, Clinical Psychology Research Laboratory, 20149 Milan, Italy

**Keywords:** Alzheimer’s disease, MCI due to AD, spatial cognition, Corsi suvra-span learning

## Abstract

Background: Spatial navigation deficits are reported as early symptoms of Alzheimer’s disease (AD) alongside episodic memory ones. The aim of the present study was to ascertain whether neuropsychological deficits of visuospatial long-term memory can predict behavioral alterations during the navigation of older adults in novel urban environments along the normal aging–dementia continuum of the Alzheimer’s type. Methods: A total of 24 community-dwelling patients with Mild Cognitive Impairment (MCI) due to AD, 27 individuals with subjective cognitive decline (SCD), and 21 healthy controls were assessed in terms of their sequential egocentric and allocentric navigation abilities by using a modified version of the Detour Navigation Test, and neuropsychologically tested by the Corsi learning suvra-span (CLSS) test. Generalized linear models were adopted to verify whether the scores obtained by the three groups in the CLSS test predicted wrong turns and moments of hesitation during the navigation task, with the results presented as topographical disorientation scores. Results: Higher scores in the CLSS test predicted fewer wrong turns (*b* = −0.05; *z* = −2.91; *p* = 0.004; net of between-groups differences) and moments of hesitation for patients with MCI due to AD (*b* = −0.14; *z* = −2.43; *p* = 0.015), and individuals with SCD (*b* = −0.17; *z* = −3.85; *p* < 0.001). Conclusions: Since the CLSS test has been reported to be a reliable measure of ecological navigational abilities in the progression towards AD dementia, we recommend its use in clinical practice and highlight implications for future research.

## 1. Introduction

Alzheimer’s disease (AD) stands as a relevant neurodegenerative disorder primarily affecting older adults and ranks as the leading cause of dementia [1]. It is characterized by a gradual decline in cognitive functions, mainly encompassing amnestic symptoms and spatial disorientation due to early decay [2].

Deficits in the free recall of objects’ locations, the temporal sequencing of landmarks, landmark recognition, route learning, and directional guidance have been revealed in patients with AD and Mild Cognitive Impairment (MCI) [3]. These deficits contribute to spatial navigation errors and may lead to topographical disorientation, even in familiar environments, as the disease progresses to its advanced stages [3,4,5].

Observing the manifestation of spatial disorientation in real-world settings may serve as a proxy variable for the early identification of incipient AD in individuals at high risk of conversion, i.e., MCI due to AD or Apolipoprotein-E (APOE)-ε4 carriers [6,7]. Furthermore, changes in walking patterns have been identified as motor features associated with cognitive impairment and dementia [8], including AD [9].

Spatial cognition comprises a complex brain system with various processes and components [10,11]. Human beings use two primary frames of reference for acquiring and organizing spatial information in memory, i.e., egocentric and the allocentric [12]. The egocentric frame defines spatial information relative to the body’s position, thus maintaining the viewpoint perspective of the navigator. Egocentric spatial representations are usually described as ‘orientation-specific’ or ‘orientation-dependent’ in relation to sequential body turns during a path-learning task [2]. By contrast, allocentric frames are not reliant on the body’s position in the space and are centered on external cues, such as landmarks [2]. Allocentric spatial representations are frequently referred to as ‘orientation-independent’ or ‘orientation-free’ [13,14] because they focus on relationships among environmental cues.

Spatial memory also plays a crucial role in encoding, storing, and retrieving spatial information. Spatial memory serves two important functions of navigational ability: route learning, which involves acquiring a new path, and wayfinding, where a navigator creates a global representation of the environment, based on landmarks used as spatial cues for orientation. These functions allow individuals to maintain topographical orientation [15] and depend on the neural activity of brain structures, including the parietal cortex and the caudate nucleus, the hippocampus, and the medial temporal lobe, respectively [16]. Moreover, executive and working memory functions have a pivotal role in spatial navigation, including tasks such as selecting appropriate strategies, identifying potential alternative approaches, maintaining navigational goals, and calculating directions and distances. Executive functioning further influences the memory system, as the effective recall of information requires strategic elaboration during both the encoding and the retrieval phases [4].

According to Baddeley [17,18], working memory consists of four distinct components. Among these, two serve as passive information maintenance systems: the phonological loop (equipped with the ability to reiterate a limited sequence of phonologically encoded information), where verbal material is stored, and the visuospatial sketchpad, where visual and spatial material is maintained. Alongside these ‘slave systems’ is the ‘Central Executive’, which allocates attentional resources for processing the information preserved in the passive maintenance systems. The most recent implementation of the model is the episodic buffer, a temporary system of storage that integrates information from both the phonological and the visuospatial subsystems with data from long-term memory [18]. Logie [19] also proposed a visuospatial working memory model that breaks down the visuospatial sketchpad into two distinct components: the visual cache and the inner scribe. The visual cache works as passive storage for visual patterns, while the inner scribe serves as an active rehearsal mechanism used during movement planning and execution. These components are closely intertwined. The inner scribe refreshes the contents of the visual cache when the maintained visual pattern becomes irrelevant. This model thus splits visual working memory, responsible for temporary visual information, and spatial working memory, which holds spatial details during the preparation and execution of a movement. The selection of the specific visuospatial sketchpad component required for a task depends on the type of information to be stored in working memory [20].

**Figure 1:**
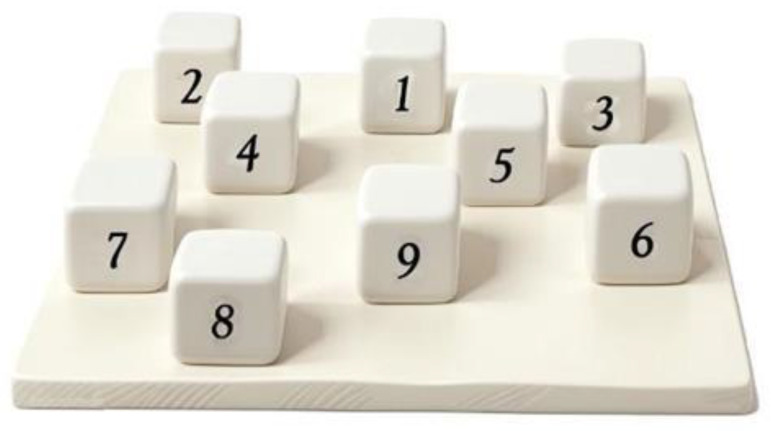
Material used in the administration of the CLSS test. The numbers appear on the side facing the examiner only.

The interface between visuospatial working memory and visuospatial episodic long-term memory is provided by the episodic buffer, employing conscious awareness as a retrieval mechanism [18]. Episodic memory is a component of long-term memory which refers to the temporospatial context of personal memories [21,22], and can be further subdivided into verbal and visuospatial components. Verbal episodic tasks involve encoding or retrieval through verbal repetition, while visuospatial episodic tasks entail material that requires interpretation through visuospatial processing [23]. Executing concrete spatial navigation tasks, such as taking a shortcut, retrieving spatial information from long-term memory, and manipulating visuospatial images to obtain new information, is crucial for spatial navigation [24]. In order to assess visuospatial long-term memory, the Corsi learning suvra-span (CLSS) test represents a neuropsychological test extensively employed in clinical practice, especially in Italian clinics and hospitals [25,26] (Figure 1).

The CLSS test may be a valid method for the evaluation of visuospatial long-term memory supporting spatial navigation [27]. In fact, to successfully navigate the urban environment, elderly people need to actively manipulate visuospatial information, integrate egocentric and allocentric abilities, and use spatial representations retrieved from long-term memory. The sectorial literature has pointed out that only some virtual tasks such as the Sea Hero Quest [28], or paper-and-pencil tasks such as the Mini Mental State Examination (MMSE) [29], the Rey–Osterrieth Complex Figure (ROCF) [30,31], and the Free and Cued Selective Reminding Test (FCSRT) [32], are associated with the spatial navigation performances of real-word navigation tasks administered to older adults with MCI and dementia [33]. However, no study to date has evaluated whether the neuropsychological examination of visuospatial long-term memory using the Corsi suvra-span procedure can effectively anticipate topographical disorientation in older adults along the normal aging–dementia continuum of the Alzheimer’s type. In line with this, our investigation sought to evaluate whether the CLSS test can predict the spatial navigation errors of community-dwelling older adults undergoing a naturalistic experimental task along the normal aging–dementia continuum of the Alzheimer’s type. In particular, we hypothesized that better performance in the CLSS task corresponds to a lower number of errors in a naturalistic spatial navigation task, effectively anticipating topographical disorientation in older adults.

## 2. Materials and Methods

### 2.1. Participants

The study included 24 patients with MCI due to AD, 27 individuals with subjective cognitive decline (SCD), and 21 healthy controls (HCs). Patients with MCI due to AD were diagnosed using the criteria from Petersen et al. [34] and Dubois et al. [35,36], while individuals with SCI were diagnosed using the criteria from Jessen et al. [37]. In addition to these participants, cognitively unimpaired older adults were enrolled through advertisements posted in social centers for senior citizens in the Milan community (Italy) and through conferences, webinars, and events concerning research pertaining to AD prevention. All participants met specific inclusion criteria, encompassing an age range between 60 and 85 years, a minimum education level of 5 years, the absence of dementia, and basic information and communication technology skills. The exclusion criteria included a history of alcohol or substance abuse, a diagnosis of neurological or psychiatric conditions, or any other medical conditions that could impact spatial navigation, such as head injury, vision loss, or motor disability. Moreover, participants with prior knowledge of the urban area used for the naturalistic task were excluded.

### 2.2. Study Design

This was an observational multi-center investigation with a regression approach.

### 2.3. Real-World Navigation Paradigm

**Figure 2:**
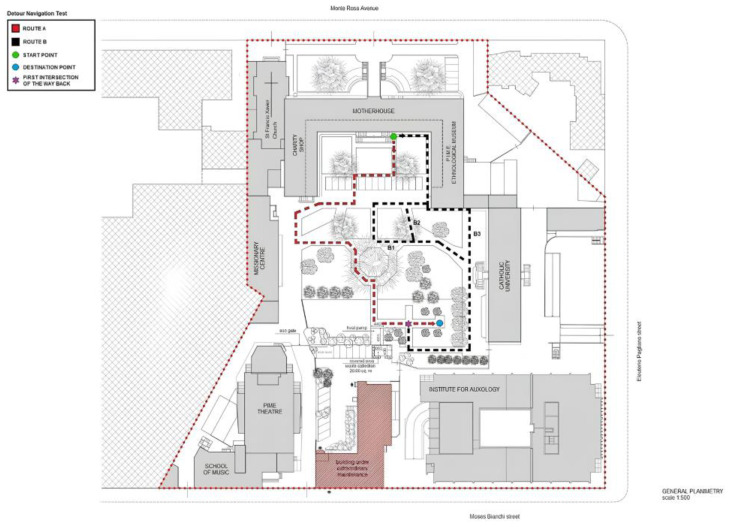
Pontificio Istituto Missioni Estere (PIME, 81 Monte Rosa Street, Milano, Italy): General planimetry and description of the Detour Navigation Test—modified version (B1, B2, and B3 represent different options for the Route B detour).

Participants engaged in a modified version of the Detour Navigation Test [28], which we designed to explore spatial alterations in patients with prodromal AD navigating unfamiliar urban environments (for a detailed description, please see [38]). The test was conducted in an ecological setting, i.e., the urban park of the Pontificio Istituto Missioni Estere—PIME (81, Monterosa Street, Milan, Italy) (Figure 2). Participants wore a sensory garment (the Howdy Senior^®^ system by ComfTech S.r.l., Monza, Italy) and were asked to complete a path from an initial start point to a designated endpoint (Route A, outward) by following an investigator-guided walk, and pay attention to all the landmarks. They were also required to perform an interfering algebraic task while walking until they reached the destination point. This task was designed to guarantee the experiment’s ecological validity and replicate the distracting elements of an urban environment. Once at the destination, participants were tested on landmarks recognition. After this task, participants had to reach the start point by retracing the same route (Route A, return) alone, with feedback provided by the experimenter for deviations from the correct path. This route-retracing task mainly represented egocentric sequential navigation supported by the recall of body turns along a path backwards. Then, the participants were asked to reach the destination point (Route B, outward) again. However, unknown to them, at the first intersection of the way back, the participants were asked to walk a different route (Route B, return) that did not overlap with Route A (return) at all. Such a cognitive/motor task required the use of an allocentric strategy based on a built cognitive representation of the urban park. For this second experimental part, feedback was not allowed. Wrong turns, measured in numbers (n.) and moments of hesitation (in seconds), were recorded during the Route A return and the Route B return and added up, and we assessed deviations from the predefined path and uncertainties, respectively (for a complete description and calculation of these two disorientation scores, please see [38]). The observational protocol for reporting behavioral data and the experimental procedure for the collection and registration of wrong turns and moments of hesitation are reported in Supplementary Materials.

### 2.4. Neuropsychological Evaluation

Prior to the ecological task, we evaluated participants at the hospital near to the PIME urban garden (The Mosé Bianchi, IRCCS, Istituto Auxologico Italiano, Milan, Italy), administering a screening of global cognition through the Montreal Cognitive Assessment (MoCA) [39]. Given that our study had a multi-center recruitment strategy, some of the participants were previously evaluated using the MMSE [40,41]. For these participants, we adopted Aiello and colleagues’ [42] conversion norms to obtain MoCA scores in order to correctly compare all groups. Participants were also evaluated to assess their affective status by using the Geriatric Depression Scale [43], and their visuospatial long-term memory abilities were assessed by the CLSS test [25].

In this neuropsychological test, nine wooden cubes (4.5 cm × 4.5 cm) are attached to a board (32 cm × 25 cm) in a standard arrangement [25]. The examiner presents a fixed sequence of eight cubes exceeding short-term memory capacity, presenting one cube every 2 s. Following each demonstration, the subject is required to exactly reproduce the sequence, and the examiner records the touched cubes considering only the first 8 cubes selected in case of iterative behavior. The sequence is repeated until the learning criterion is fulfilled (i.e., three consecutive correct repetitions produced by the examinee) or up to a maximum of 18 trials. After a 5 min interval, the examinee is then asked to reproduce the sequence without the experimenter’s demonstration (i.e., the delayed recall score). The re-enactment of the sequence without a new presentation is assessed independently from the preceding 18 trials [25,26]. The test takes about 15 min to be administered.

The quantitative evaluation of performance in the CLSS test takes into account all cubes that were touched in the correct order, as well as their possible combinations (i.e., 0 = 0.36; 2 = 0.30; 3 = 0.52; 4 = 0.74; 5 = 0.96; 6 = 1.18; 7 = 1.40; 8 = 1.62; 2 + 2 = 0.53; 2 + 3 = 0.75; 2 + 4 and 3 + 3 = 0.97; 2 + 5 and 3 + 4 = 1.19; 2 + 6, 3 + 5, and 4 + 4 = 1.41; 2 + 2 + 2 = 0.76; 2 + 2 + 3 = 0.99; 2 + 3 + 3 = 1.21, 2 + 2 + 2 + 2 = 1). The learning score (LS) was calculated by adding up all the sub-scores obtained from the 18 trials, ranging from 0 to 26.16. This final raw score was then appropriately adjusted based on age, education, and gender [25].

### 2.5. Statistics

Scores for wrong turns and moments of hesitation were checked for normality by computing skewness and kurtosis (judged by indexing non-normal distributions if >|1| and |3|), respectively [44] and by observing histograms and quantile–quantile plots. As both these variables proved to be heavily right-skewed and over-dispersed, the ecological validity of the CLSS test was separately tested against wrong turns and moments of hesitation via generalized linear models underlying a negative binomial distribution [45]. *Group* was also entered as a factor within these two models, with a CLSS*Group interaction term also fitted in order to test whether the predictive capability of the CLSS test differed across healthy controls (HCs), SCD individuals, and MCI patients. The post hoc comparisons regarding *Group* effects were Bonferroni-corrected, whilst the CLSS*Group interaction was decomposed via simple slope analyses. The analyses were run via Jamovi 2.3 (The Jamovi project, 2023).

## 3. Results

The sociodemographic, clinical, neuropsychological, and behavioral measures from the modified version of the Detour Navigation Test and comparisons among groups are all reported in Table 1.

The three groups were balanced for age (*F*(2, 43.45) = 1.38, *p* = 0.263), gender (χ^2^(2) = 3.38; *p* = 0.185), and education (*F*(2, 45.33) = 0.91, *p* = 0.408). The GDS scores differed significantly across groups (*F*(2, 41.29) = 6.41, *p =* 0.004), being lower in HCs when compared to individuals with both SCD (*p* = 0.031) and MCI (*p* = 0.020). The comparison between MCI and SCD patients was not significant for depression (*p* = n.s.). A significant discrepancy was also detected in the MoCA scores (*F*(2, 43.90) = 23.24, *p* < 0.001), with MCI patients performing worse than both SCD patients (*p* <.001) and HCs (*p* = 0.001), and SCD patients performing better than HCs (*p* = 0.003).

A similar pattern was detected as for the CLSS test (*F*(2, 45.33) = 21.36, *p* < 0.001), with MCI patients reporting lower scores on this test when compared to both individuals with SCD (*p* < 0.001) and HCs (*p* < 0.001), and a trend was detected towards lower scores in SCD patients when compared to HCs (*p* = 0.069). Significant between-group differences were also detected with regard to wrong turns (χ^2^(2) = 21.82; *p* < 0.001) and moments of hesitation (χ^2^(2) = 19.24; *p* < 0.001), with MCI patients performing worse than SCD patients (wrong turns: *p* < 0.001; moments of hesitation: *p* < 0.001) and HCs (wrong turns: *p* < 0.001; moments of hesitation: *p* < 0.001) on both measures. The other comparisons between individuals with SCD and HCs were not significant for these disorientation scores (*p* = n.s.).

**Figure 3:**
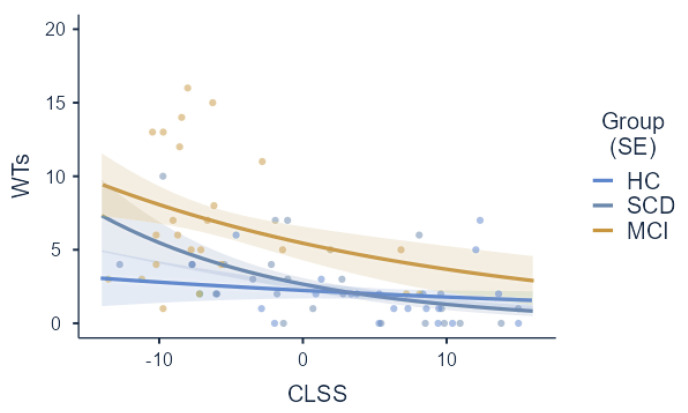
Prediction of CLSS scores for wrong turns split by group. **Notes:** CLSS = Corsi learning suvra-span; WTs = wrong turns; HCs = healthy controls; SCD = subjective cognitive decline; MCI = Mild Cognitive Impairment. Slopes are represented along with respective *SE*s.

The generalized linear model focusing on wrong turns revealed that higher CLSS scores predicted lower wrong turns (*b* = −0.05; *z* = −2.91; *p* = 0.004), detected as the net of between-group differences (χ^2^(2) = 9.28; *p* = 0.010) which were calculated in the post hoc comparisons, with MCI patients reporting more wrong turns than both individuals with SCD (*p* = 0.020) and HCs (*p* = 0.019). Regarding variance, no significant CLSS*Group interaction (χ^2^(2) = 1.96; *p* = 0.375) was detected (Figure 3).

**Figure 4:**
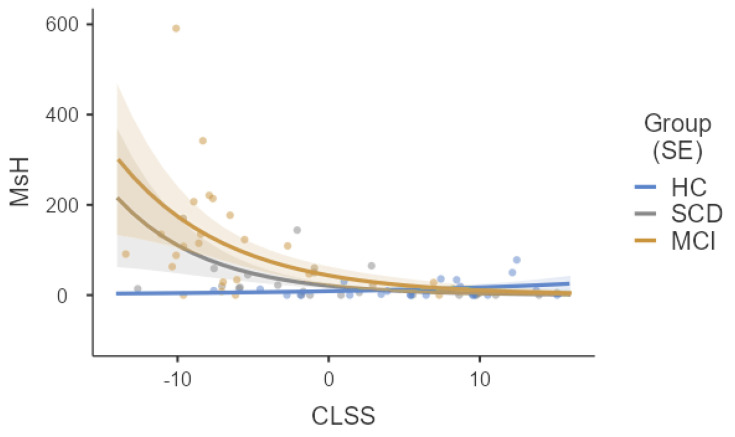
Prediction of CLSS scores for moments of hesitation split by group. **Notes:** CLSS = Corsi learning suvra-span; WTs = wrong turns; HCs = healthy controls; SCD = subjective cognitive decline; MCI = Mild Cognitive Impairment. Slopes are represented along with respective *SE*s.

Similarly, higher CLSS scores predicted lower scores for moments of hesitation as well (*b* = −0.08; *z* = −2.60; *p* = 0.009). Within this model, whilst *Group* did not yield a significant effect *per se* (χ^2^(2) = 5.55; *p* = 0.062), a significant interaction was detected between *Group* and the CLSS test (χ^2^(2) = 9.74; *p* = 0.008). A posteriori, simple slope analyses (Figure 4) revealed that CLSS scores were predictive of moments of hesitation in SCD (*b* = −0.17; *z* = −3.85; *p* < 0.001) and MCI patients (*b* = −0.14; *z* = −2.43; *p* = 0.015), but not in HCs (*b* = 0.07; *z* = 1.15; *p* = 0.249).

## 4. Discussion

Our study aimed to determine whether the CLSS test can predict spatial navigation errors in novel urban environments for community-dwelling older adults from normal aging to AD. We documented that this test was able to predict spatial navigation errors such as wrong turns beyond participants’ cognitive profiles and moments of hesitation for individuals with SCD and patents with MCI due to AD.

The results from this study are in line with our previous investigation [46] and represent a step forward. In fact, patients with MCI due to AD also showed a higher number of wrong turns and more moments of hesitation than individuals with SCD and HCs, as we already noticed [46]. Moreover, they reported lower performance than the other two groups in global cognitive screening (MoCA) and CLSS, as expected, as well as in depression (GDS), given that depressive symptoms in MCI appear to be predictors for progression to AD dementia. Individuals with SCD presented with better performance in MoCA than HCs, probably because cognitive effort in sustaining the global screening test was influenced by greater activation aimed at confirming the absence of the neurological deficit, with patients already aware of the integrity of their cognitive profile before the experiment.

Spatial memory relies on brain structures that are particularly susceptible to both normal aging and degenerative dementia [15]. The hippocampus, the fronto-parietal network, and the temporal lobe collaborate in sustaining spatial memory [15]. In AD, neurodegeneration primarily affects the hippocampus, leading to anterograde amnesia as an early symptom. However, the hippocampus is also crucial for visuospatial processes, sustaining both sequential egocentric abilities and allocentric abilities, as we already discussed for patients with MCI due to AD undergoing the modified version of the Detour Navigation Test [46]. Additionally, early targets in AD are the temporal-parietal areas, which are related to spatial and visuo-constructive abilities. The evidence on progressive damage to the neural circuits supporting spatial memory in AD suggests that subtle impairments in spatial navigation are already present in MCI due to AD and possibly serve as a potential biomarker for the risk of developing AD dementia [14].

**Figure 5:**
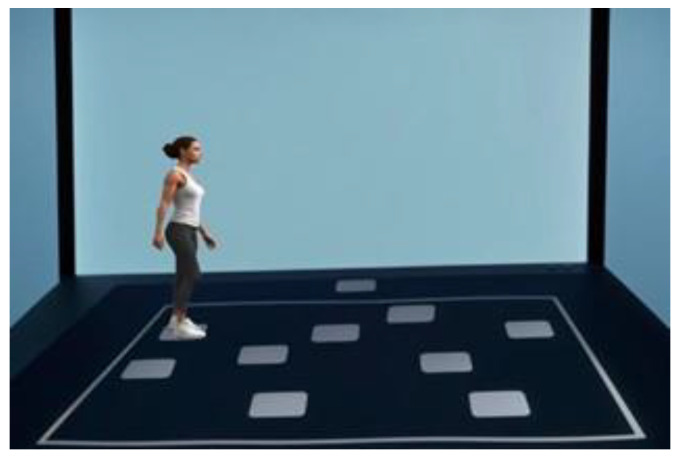
The equipment of the Walking Corsi test.

A large-scale variant of the CLSS test, the Walking Corsi test [47], has also been developed to specifically assess navigational memory proficiency during the execution of a predefined path (Figure 5). This test is conducted in a featureless room measuring 5 m × 6 m, with walls entirely concealed by curtains obscuring any external landmarks. Nine black squares (30 cm × 30 cm) are positioned on a light-grey carpet measuring 2.50 m × 3.00 m, arranged in a manner identical to the scaled position and relative spatial layout observed in the CLSS test. The administration of the Walking Corsi test is identical to that of the CLSS test, with the examiner walking on the carpet and pausing for 2 s on each square while presenting the sequences. The Walking Corsi test has been employed in clinical settings and has been proven to be a valid instrument for evaluating individuals in the initial phases of AD, given its sensitivity in the early detection of topographical deficits [48].

Whereas the CLSS test can be considered a gold standard for the assessment of visuospatial long-term memory, little research has been conducted into its potential use for testing spatial navigation abilities in real-world setting [47,48]. We documented that the CLSS test can predict deviations from a predefined route expressed as wrong turns in the elderly (net of group belonging) and that it is a valid measure to anticipate moments of hesitation for SCD individuals and patients with MCI due to AD, probably because they represent the preclinical and premorbid phase of AD dementia in which people start demonstrating spatial disorientation, being partially unaware of their whereabouts and unable to navigate to an intended unfamiliar location [49]. These findings represent a promising scenario in terms of CLSS ecological validity, defined as the ability of performance-based measures of cognition to accurately represent patients’ behavior in real-world settings, which is referred in the literature as ‘veridicality’ [50].

Neuropsychological tests are often biased because they are based on laboratory tasks with poor ecological validity. As a result, evaluating ecological validity typically involves retrospectively assessing how neuropsychological tests can really reflect real-life cognitive functioning. This is usually explored by examining the correlation between performance-based cognitive measures and reports from proxies about an individual’s cognitive abilities in everyday life [51]. Considering these assumptions, we demonstrated the ecological validity of the CLSS test in terms of veridicality, specifically focusing on assessing its capacity to predict spatial navigation alterations along the physiological aging–AD continuum.

However, our study had some limitations. While neuropsychological tests such as the CLSS test continue to contribute to the diagnosis of some neurological conditions, such as detecting early stages of AD (e.g., [52]), they often reflect the activity of multiple neural circuits, which may limit their precision in locating brain damage [53]. Moreover, the veridicality approach has been subject to criticism because a direct comparison between traditional neurocognitive tests and functional performance evaluated through behavioral observation is not often evident [53]. By using the Italian norms from Aiello and colleagues [42], we converted the MMSE scores into MoCA scores for those participants who were not assessed by the latter instrument, since our study was a multi-center investigation recruiting participants from three sites [38]. The three groups were not perfectly balanced either, even if they were well matched in terms of age, education, and gender. The spatial navigation performance of elderly people may be also affected by anxiety. Beyond depression, which we measured, anxious symptoms may influence the subjective perception of spatial navigation abilities in elderly people [54], thus representing a relevant variable that should be considered in such a specific research line. In the design of our study [46], the participants were administered different neuropsychological assessment tools for the evaluation of visuospatial short-term memory, too, such as the Corsi block-tapping test [55,56,57] to evaluate the backward and the forward spans, or the ROCF immediate recall test [58,59] to evaluate visuo-constructional ability and visual memory. This procedure did not allow the researchers to have a unique measure of short-term visuospatial memory, which would have been useful for comparison with the long-term component. Finally, given that visuospatial short-term memory is also involved in sustaining spatial navigation, we could have also used this variable to forecast the spatial navigation abilities critical to the everyday independence of elderly people in their communities, especially if affected by AD-related cognitive symptoms. Research in the real world poses a number of challenges, due to the need to ensure that everyone has identical conditions to optimize task design [60]. Potentially disturbing factors in the real-word settings, such as weather conditions (e.g., light or temperature), background noise, the presence of individuals who need to move away from the urban circuit prior to the experiment, or landmarks of the research working setting that need to be rearranged before carrying out the naturalistic test, were difficult and time-consuming to control. To this end, the researchers adopted all the possible countermeasures to eliminate confounding factors.

Carefully examining whether neuropsychological tests are appropriate for a particular purpose represents a crucial feature of clinical research. Choosing valid instruments for the assessment of the premorbid phase of dementia is challenging for clinicians, given that they should provide valuable information about the personal autonomy of older adults in their everyday environments. Spatial navigation is a complex cognitive skill that is necessary for the everyday functioning of elders, increasing independence and reducing caregivers’ burden. This line of research is particularly hindered by some methodological issues, such as difficulty in translating performance in standardized neuropsychological testing into real-world abilities, the correct measurement of the real-world functioning of older adults, several aging-related factors (e.g., maintaining cognitive reserve) that may play a mediating role, and the influence of confounding variables related to the natural environment. To this end, our investigation significantly contributed to the further development of scientific knowledge in the field of the neuropsychological assessment of the premorbid phase of Alzheimer’s dementia.

## 5. Conclusions

Spatial navigation may be impaired early in the course of AD and represents a marker of clinical dementia progression. Patients with AD frequently lose their way in familiar and unfamiliar environments, showing topographical disorientation. We reported the CLSS test as a potential reliable measure of ecological navigational abilities in elderly people along the normal aging–dementia continuum of the Alzheimer’s type. For this reason, we recommend its use in routine clinical practice when older adults and/or their informant caregivers complain about spatial disorientation symptoms during clinical interviews, and in rehabilitation intervention as a fundamental neuropsychological tool for pre/post-tests.

## Figures and Tables

**Table 1:** Participants’ sociodemographic, clinical, neuropsychological, and behavioral measures.

	HCs	SCD	MCI	*p*
*N*	21	27	24	-
Sex (male/female)	9/12	12/15	16/8	0.185 ^c^
Age (yrs.)	62.9 ± 10.7 (28–82)	54.1 ± 10.9 (24–86)	54.1 ± 10.9 (24–86)	n.s. ^d^
Education (yrs.)	12.7 ± 3.8 (5–18)	13.2 ± 4.0 (5–22)	13.2 ± 4.0 (5–22)	n.s. ^d^
GDS	12.7 ± 3.8 (5–18)	13.2 ± 4.0 (5–22)	13.2 ± 4.0 (5–22)	SCD > HCs (*p* = 0.031) ^d^ MCI > HCs (*p* = 0.020) ^d^
MoCA (adjusted scores) ^a^	12.7 ± 3.8 (5–18)	13.2 ± 4.0 (5–22)	13.2 ± 4.0 (5–22)	SCD > HCs (*p* = 0.003) ^d^ MCI < HCs (*p* = 0.001) ^d^ MCI < SCD (*p* < 0.001) ^d^
CLSS (adjusted scores) ^b^	12.7 ± 3.8 (5–18)	13.2 ± 4.0 (5–22)	13.2 ± 4.0 (5–22)	SCD > HCs (*p* = 0.069) ^d^ MCI < HCs (*p* < 0.001) ^d^ MCI < SCD (*p* < 0.001) ^d^
WTs (n.)	12.7 ± 3.8 (5–18)	13.2 ± 4.0 (5–22)	13.2 ± 4.0 (5–22)	MCI > HCs (*p* < 0.001) ^e^ MCI > SCD (*p* < 0.001) ^e^
MsH (s)	12.7 ± 3.8 (5–18)	13.2 ± 4.0 (5–22)	13.2 ± 4.0 (5–22)	MCI > HCs (*p* < 0.001) ^e^ MCI > SCD (*p* < 0.001) ^e^

Notes: HCs = heathy controls; SCD = subjective cognitive decline; MCI = Mild Cognitive Impairment; GDS = Geriatric Depression Scale; MoCA = Montreal Cognitive Assessment; CLSS = Corsi learning suvra-span; WTs = wrong turns (number of, n.); MsH = moments of hesitation (in seconds, s); ^a^ [39] MoCA normative dataset; ^b^ [26] CLSS normative dataset; ^c^ χ^2^-statistic for independent samples; ^d^ Tukey-adjusted *p*-values for *F*-statistic; ^e^ Dwass–Steel–Critchlow–Fligner-adjusted *p*-values for Kruskal–Wallis χ^2^-statistic.

## Data Availability

The datasets presented in this article are not readily available due to technical limitations. Requests to access the datasets should be directed to davide.cammisuli1@unicatt.it. We used the AI software Leonardo.ai (Basic version) to create the Corsi suvra-span test and the surface and volume rendering of the Corsi Waling Test, including the character. However, we retain the right to reproduce and distribute them as our intellectual property. According to Section 3 of the Leonardo.ai Terms of Service, the user of the platform retains ownership of all input he or she provides to the services and receives all rights, titles, and interest in the output generated and returned by the services based on the input. The content over which these rights are benefited from may be used for commercial purposes. Users are granted a worldwide, non-exclusive, royalty-free license to access the resources publicly available through the service and to use those resources (including reproduction, distribution, modification, display, and performance) as permitted by the service.

## Supplementary Materials

The following supporting information can be downloaded at: https://www.mdpi.com/article/10.3390/jpm14050539/s1, Experimenter’s Observation Protocol of Behavioral Data during the Naturalistic Spatial Navigation Task.
