# Supplementary material for: Behavioral Disorders of Spatial Cognition in Patients with Mild Cognitive Impairment Due to Alzheimer’s Disease (The BDSC-MCI Project): Ecological Validity of the Corsi Learning Suvra-Span Test
Source: J Pers Med. 2024 May 17;14(5):539. doi: 10.3390/jpm14050539 (PMC11122154; doi:10.3390/jpm14050539)
Supplement: Supplementary file 1 [file jpm-14-00539-s001.zip › jpm-2980876-supplementary.pdf]

Supplementary Material. Experimenter's Observation Protocol of Behavioral Data during the Naturalistic Spatial Navigation Task

1. The volunteer patient wears the Howdy Senior garment;
2. Correct size is chosen, electronics are attached but not switched on yet, and electrodes are moistened (profusely, with moistened paper until droplets fall).
   Women are required to take off their bras and both women and men will now wear the monitoring unit on bare skin.
3. Electronics are switched on through a quick 1 s tap. The device will start flashing red and green simultaneously. As soon as the device flashes blue, electronics are ready to begin monitoring. At this point the volunteer is required to sit on a chair as the start and end of the measuring phase of physiological parameters is noted down, approximately 5 min. The resting monitoring phase will be named '**REST state**'. A stopwatch is employed to enter data.
   Start: ___________; End: ____________; Exact Counting: _________________
4. **ROUTE A, outward:** Once the monitoring unit is put on, the participant is taken out into the garden and required to stand at the starting point of the route. After connecting the smartphone to the internet via hotspot and with the Howdy Senior physiological data monitoring and the Howdy Senior Aptive gait analysis Apps, he/she is positioned standing on his/her feet firmly at the starting point of the route and given the following instruction: "*I am now going to show you the path we will take around this garden. Please pay attention to what we will do and to the landmarks we shall find along the way. When I say 'GO', you will start walking. At certain stop along the way, I will ask you to perform a simple algebraic operation but please remember that you must not stop walking. I will stop you once we have reached our destination*".
5. We then start our stopwatch (thus marking the beginning of the route). Wait a few seconds for the application (Aptive) to geolocalise the participant. Then click on 'REC' to record the route. Once we have arrived at the end of the Route A, the participant is asked to sit on the bench and rest. Then, click 'STOP' to mark the end of the Active app. Look at the stopwatch and record the time taken.
   Start: ___________; End: ____________; Exact Counting: _________________

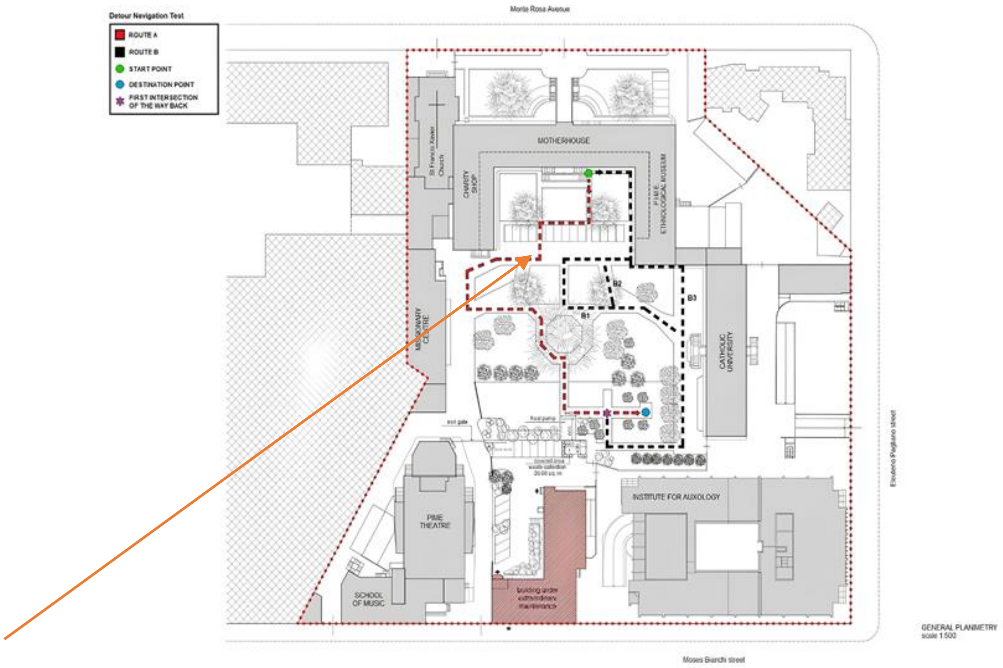

Starting point of the distracting algebraic task.

6. The patient is asked to perform the landmark recognition task: together we browse through the pictures of the landmarks collected inside the portable folder. We then give the following instruction: *"I am now going to show you pictures of elements you may have encountered during this tour. You will have to tell me whether you have seen these elements or whether you have never seen them"*.

**LANDMARK**	**RECOGNIZED (hits)**	**Distractors**
A1		
A2		
B1		
B2		
C1		
C2		
D1		
D2		
E1		
E2		
F1		
F2		

Please, put a tick in the right column.

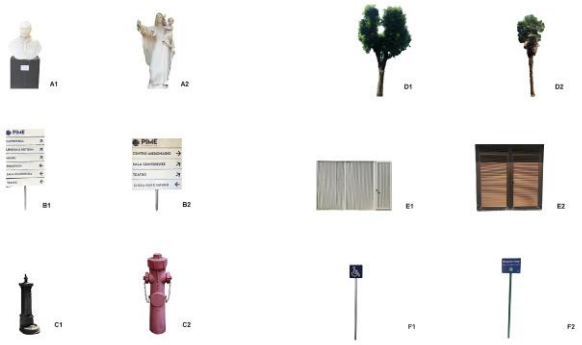

7. **ROUTE A, return:** Once the recognition task is done, we ask the participant to get up from the bench and give him/her a new instruction: *"Now go back to the starting point by retracing exactly the same route I previously showed you. Are you ready? Go!"*. We start the Howdy Senior and Aptive Apps again, as well as the stopwatch.

Start: ___________; End: ____________; Exact Counting: _________________

PLEASE NOTE: ***If we see that the participants make the wrong choice in the Route A return, you have to correct him/her to allow a correct registration of the Route A backwards.***

The table below should be filled in for this purpose:

- **WRONG TURNS (WTs)** are when the participant makes deviations from the path previously taken. In this case, when he/she makes a mistake it is immediately recorded as a *wrong turn* and the subject is told: *"This was not the correct route, try and remember the correct one!"*.
- **MOMENTS OF HESITATION** (MsH) are when you notice that the participant slows down to a halt, looks around to reorient and/or asks the experimenter what the correct route is. You have to record it, immediately, and, while checking on our stopwatch, you mark the exact time of MH start and end, so that you know how long it lasted.

ROUTE A, return—WTs.

WTs	Sequential numbering	Patient has<br>A. deviated from Route A;<br>B. trodded a non-viable path.

Please, put a tick in the first column.

ROUTE A, return—MsH.

MsH	Sequential numbering	Exact time in the route sequence when the MH has occurred	Duration of the hesitation (minutes, seconds and hundredths)

Please, put a tick in the first column.

8. **ROUTE B, outward:** Once the participant has returned to the starting point, you then move onto 'ROUTE B'. The subject is taken back along the same route as the Route A, here you must record the start/end time and use the Howdy Senior and Aptive apps, again. You give the following instruction: *"Now we will walk the same route as before until we arrive at the destination point, that is the bench where you previously sat to rest and recognize landmarks. Are you ready? Go!"*.

   Start: ___________; End: ____________; Exact Counting: _________________

9. **ROUTE B, return:** Once arrived at the bench at the end of the route, you ask the participant to sit down and rest for a moment. You stop the Apps and the stopwatch. You then take him/her to the first useful intersection (the one closest to the bench) and tell him/her: *"Now you will have to go back to the starting point, but cannot walk the same route as before, you will have to find an alternate route that does not overlap in any way with the route we took before. You may only walk on paved paths and gravel, you cannot walk on the grass. Are you ready? Go!"*. At this point, we start the Howdy Senior and Aptive Apps and the smartwatch again. You record the start and end of the route.

   Start: ___________; End: ____________; Exact Counting: _________________

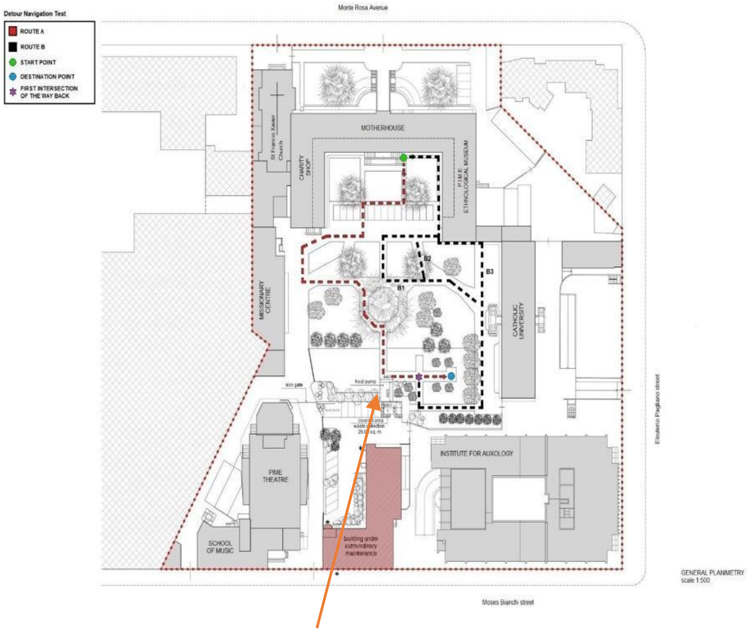

First intersection of the way back.

10. You also record the return route travelled (B1, B2 or B3). Route travelled: ____________

PLEASE NOTE(i): You have to mark WTs for Route B return as well, which in this case are the overlaps with the Route A return, and MsH in the same way as for the Route A return. However, during the Route B return, unlike the Route A, if the participant makes a mistake, you have to not correct him/her, but let participant goes his/her way. If participants ask for suggestions, you reply that you cannot provide an answer.

PLEASE NOTE(ii): In situations of manifest cognitive impairment, the maximum length for a MH allowed to any participant is 5 min. Exclusively in this case, while recording the MH, please put him/her back on the correct track (either B1, B2 or B3).

We then close the Howdy Senior and the Aptive Apps and click on "GENERATE REPORT" on the experimenter's smartphone, using the corresponding button. The end time of the task is already entered by default. Please check that the start time corresponds to the initial rest state.

ROUTE B, return—WTs

WTs	Sequential numbering	Patient has<br>A. overlapped with Route A;<br>B. trodded a non-viable path.

Please, put a tick in the first column.

ROUTE B, return—MsH

MsH	Sequential numbering	Exact time in the route sequence when the MH has occurred	Duration of the hesitation (minutes, seconds and hundredths)

Please, put a tick in the first column.
